# A Dual-Sensitizer Strategy for Enhanced Photocatalysis by Coupling Perylene Tetracarboxylic Acid and Copper Phthalocyanine Tetracarboxylic Acids on TiO_2_

**DOI:** 10.3390/ma18204715

**Published:** 2025-10-14

**Authors:** Alina Raditoiu, Florentina Monica Raduly, Maria Grapin, Radu Claudiu Fierascu, Cristian-Andi Nicolae, Bogdan Trica, Valentin Raditoiu

**Affiliations:** 1 National Institute for Research & Development in Chemistry and Petrochemistry—ICECHIM, 202 Splaiul Independentei, 060021 Bucharest, Romania; coloranti@icechim.ro (A.R.); monica.raduly@icechim.ro (F.M.R.); maria.grapin02@gmail.com (M.G.); fierascu.radu@icechim.ro (R.C.F.); ca_nicolae@yahoo.com (C.-A.N.); bogdan.trica@icechim.ro (B.T.); 2 Faculty of Chemical Engineering and Biotechnology, National University of Science and Technology, Politehnica Bucharest, 1–7 Gh. Polizu Street, 011061 Bucharest, Romania

**Keywords:** photocatalyst, semiconductor dyes, hybrid materials, perylene dyes, metalphthalocyanine derivatives

## Abstract

Titanium dioxide (TiO_2_) is a widely used photocatalyst, yet its activity is limited to ultraviolet light due to its large band gap. To extend absorption into the visible spectrum, this study developed a dual-sensitizer strategy by coupling perylene tetracarboxylic acid (PTCA) and copper phthalocyanine tetracarboxylic acid (CuPcTC) onto TiO_2_. Both dyes were selected for their strong visible light absorption, photostability, and efficient charge transfer properties. Hybrid photocatalysts were prepared via an ultrasonication–coprecipitation method and incorporated into coatings. Optical, morpho-structural, thermal, and electrochemical methods were used to characterize the hybrid photocatalysts, while photocatalytic performances were evaluated by UV–Vis spectroscopy, hydroxyl radical generation, and Methylene Blue degradation under simulated solar light. The dual-sensitized TiO_2_ composites exhibited broadened absorption across 400–750 nm, effective charge separation, and stable radical generation. Among the tested samples, the PTCA–CuPcTC hybrid (P3) demonstrated the highest activity, achieving efficient degradation of Methylene Blue with sustained performance over repeated cycles. Characterization confirmed uniform distribution of sensitizers, high crystallinity, and adequate thermal stability. These findings indicate that combining PTCA and CuPcTC provides synergistic benefits in light harvesting, charge transfer, and durability. The dual-sensitizer approach offers a promising route for visible-light-responsive photocatalysts in environmental remediation.

## 1. Introduction

Titanium dioxide (TiO_2_) remains one of the most widely used semiconductor photocatalysts, owing to its chemical stability, low toxicity, and strong oxidative power. However, its wide band gap restricts photoactivation to the UV region, which accounts for only about 5% of the solar spectrum [1]. Strategies to enhance TiO_2_’s photocatalytic performance include doping with metals or non-metals, coupling with other semiconductors, or functionalizing its surface with organic sensitizers [2,3,4,5]. These modifications improve visible light absorption, reduce electron–hole recombination, and increase interfacial charge transfer efficiency, thereby significantly boosting the degradation rates of contaminants in water [6,7]. As a result, engineered TiO_2_-based photocatalysts offer a promising, sustainable approach for effective water treatment and environmental remediation [8,9,10,11].

Over the past decade, TiO_2_-based hybrid photocatalysts have evolved from simple semiconductor heterojunctions to increasingly sophisticated multi-component systems that combine TiO_2_ with dyes, narrow-bandgap semiconductors (e.g., CdS, PbS), co-catalysts, carbon materials, conductive polymers or MOFs—each improving visible light absorption, charge separation, or surface reactivity [12,13]. Reviews going back to early roadmaps [14] already highlighted the promise of sensitization and heterojunctions for expanding TiO_2_’s spectral response and overcoming fast electron–hole recombination [15,16]. Later works, including state-of-the-art reviews, detail how TiO_2_/MOF composites or semiconductor–semiconductor hybrids can dramatically enhance pollutant degradation, CO_2_ conversion, or hydrogen evolution by facilitating interfacial charge flow and tailored light harvesting [17].

Within this landscape, the “dual-sensitizer” approach represents a strategic convergence of visible light sensitization and heterojunction design. By anchoring two distinct light absorbers (e.g., CdS and PbS, or dye plus semiconductor) onto TiO_2_, dual-sensitization constructs a ternary heterostructure that broadens the solar absorption range and promotes directional charge transfer through Z-scheme or type-II cascades—yielding photocatalytic performance significantly superior to single-sensitizer counterparts [18]. For example, emerging work [19,20] demonstrates that such dual-sensitized hybrids deliver enhanced degradation rates, hydrogen evolution and pollutant mineralization by synergistically combining complementary band alignment and charge separation pathways. Thus, the dual-sensitizer approach stands at the intersection of visible light sensitization and nanostructured heterojunction engineering—a powerful progression in the evolving field of TiO_2_-based hybrid photocatalysts [21,22,23]. Sensitization with organic dyes is a common strategy to utilize visible light [24].

Copper phthalocyanine tetracarboxylic acids (CuPcTC), a metallophthalocyanine derivative, is a promising sensitizer due to its strong absorption in the visible region, excellent chemical stability, and efficient photoinduced electron transfer properties. CuPcTc molecules have a highly conjugated macrocyclic phthalocyanine structure with peripheral carboxyl groups, which enable strong anchoring onto the TiO_2_ surface via covalent bonding or coordination interactions [25,26]. When exposed to visible light, CuPcTc absorbs photons and undergoes excitation from its ground state to an excited singlet or triplet state. These excited electrons are subsequently injected into the conduction band of TiO_2_, where they participate in redox reactions such as the degradation of pollutants, water splitting, or hydrogen evolution [27]. Meanwhile, CuPcTc is regenerated through electron donation from surrounding species, thus completing the photocatalytic cycle.

The TiO_2_/CuPcTc system benefits from several synergistic effects. The broad visible light absorption of CuPcTc (typically spanning 600–750 nm) complements TiO_2_’s UV absorption, significantly broadening the active spectrum [28,29]. Additionally, the efficient charge separation at the CuPcTc–TiO_2_ interface reduces electron–hole recombination, improving photocatalytic efficiency. Studies have shown that CuPcTc-sensitized TiO_2_ exhibits enhanced degradation of organic dyes and phenolic compounds under visible light compared to bare TiO_2_ [30].

Achieving optimal CuPcTc loading is critical—insufficient coverage limits light absorption, while excessive coverage may block active sites or hinder charge transfer. Additionally, the long-term photostability of CuPcTc on TiO_2_ under continuous irradiation needs improvement, as dye photodegradation can reduce activity over time. To address these issues, recent studies have explored co-sensitization with other dyes, heterojunction formation, and protective overcoatings to stabilize the dye–semiconductor interface [31,32,33].

Among various organic sensitizers, perylenetetracarboxylic acid (PTCA) and its derivatives have attracted attention due to their excellent photochemical stability, strong visible light absorption, and efficient electron transfer properties [34].

PTCA possesses a planar conjugated structure and multiple carboxyl groups that enable strong anchoring to the TiO_2_ surface through chemisorption. Upon visible light illumination, PTCA absorbs photons and becomes photoexcited. The excited electrons from PTCA are rapidly injected into the conduction band of TiO_2_, while the oxidized PTCA molecules can be regenerated through interactions with electron donors in the reaction environment. This photoinduced charge separation significantly enhances the visible light activity of TiO_2_, enabling photocatalytic reactions such as pollutant degradation, water splitting, and organic synthesis under sunlight or artificial visible light sources [35,36,37].

The TiO_2_/PTCA system demonstrates several advantages over bare TiO_2_. First, the sensitizer extends light absorption into the 400–600 nm range, better matching the solar spectrum. Second, PTCA facilitates interfacial charge transfer, reducing recombination losses. Finally, the structural compatibility between TiO_2_ and PTCA, enabled by the carboxyl functional groups, ensures stable and efficient interfacial electronic coupling.

Co-sensitization involves adsorbing two or more complementary dyes on TiO_2_ surfaces to extend light absorption across visible and near-infrared ranges, while mitigating aggregation and recombination issues that often plague single-dye systems. Early work demonstrated triple-dye co-sensitization, achieving over 70% photocurrent action across 400–700 nm and a DSSC efficiency of 6.5% [38].

Both CuPcTc and PTCA are planar, π-conjugated organic molecules with carboxylic acid anchoring groups that enable strong binding to TiO_2_ surfaces [39,40]. Individually, each sensitizer extends TiO_2_’s absorption into the visible region—CuPcTc primarily absorbs in the 600–750 nm region (Q-band), while PTCA absorbs strongly between 400–550 nm. By coupling these two sensitizers, it is theoretically possible to achieve broader solar absorption, covering the entire visible spectrum, synergistic charge separation, where one dye may act as a donor or acceptor relative to the other, enhancing charge transfer kinetics, and improved photostability, as electron/hole exchange between dyes could mitigate photobleaching.

## 2. Materials and Methods

### 2.1. Materials

Titanium dioxide (anatase, ≥99%, Sigma-Aldrich, St. Louis, MO, USA), Copper phthalocyanine tetracarboxylic acids (CuPcTC) was obtained by us as it was described elsewhere [41], tetrabutylammonium hydroxide (TBAOH) solution (40 wt% in water, Merck, Darmstadt, Germany), Methylene Blue (MB, ≥97%, Sigma-Aldrich, St. Louis, MO, USA), Terephthalic acid (TA, 98%, Sigma-Aldrich, St. Louis, MO, USA), 2-Hydroxyterephthalic acid (HTA, 97%, Sigma-Aldrich, St. Louis, MO, USA), Nitrotetrazolium Blue chloride (NBT, 90%, Merck, Darmstadt, Germany), Perylene-3,4,9,10-tetracarboxylic dianhydride (PTCDA, 97%, Merck, Darmstadt, Germany), Isopropanol (iPrOH, 99.9%, Chimreactiv, Bucharest, Romania), Aqua-based styrene-acrylic paint (Chimcolor Grup, Bucharest, Romania) and deionized water were used as received without further purification.

### 2.2. Synthesis of Hybrid Photocatalysts

A quantity of 1 g TiO_2_ (anatase) was dispersed in 10 mL isopropanol using ultrasonication at a maximum temperature of 35 °C. Subsequently, a quantity of 0.01 g PTCDA was dissolved in isopropanol containing TBAOH aqueous solution, by heating at 80 °C for 1 h in order to obtain 25 mL iPrOH solution (1 mM) of PTCA as TBA salt. In the same manner was prepared 25 mL iPrOH solution (1 mM) of CuPcTC as TBA salt. The dispersion of TiO_2_ was further ultrasonicated, and at a maximum temperature of 45 °C, certain volumes of PTCA and/or CuPcTC were added according to Table 1. Ultrasonication continued, and the temperature was raised to a maximum of 55 °C, after which the pH was adjusted to 7.5 using a 10 wt.% aqueous nitric acid solution. The resulting hybrid materials were separated by centrifugation and filtration, and then dried in a drying oven at 125 °C for 2 h.

### 2.3. Structural and Morphological Characterization

The samples were structurally characterized using a combination of FTIR spectroscopy, XRD analysis, and electron microscopy. FTIR spectra were recorded in the 400–4000 cm^−1^ range with a JASCO FT-IR6300 instrument (Jasco International Co., Ltd., Tokyo, Japan), equipped with a DRIFT EasiDiff accessory (Pike Technologies Inc., Fitchburg, WI, USA) allowing the identification of functional groups and chemical bonding.

Crystalline structure was examined by X-ray diffraction (XRD) in the 2θ range of 2–90° using a 9 kW Rigaku SmartLab diffractometer (Rigaku Corporation, Tokyo, Japan), operated at 45 kV and 200 mA with Cu Kα radiation (λ = 1.54059 Å) in a 2θ/θ scanning mode. The obtained diffractograms were analyzed and interpreted with PDXL software (v. 2.7.2.0), using phase identification by comparison with the ICDD database.

The morphology of the crystals produced by co-precipitation was investigated using a scanning electron microscope (SEM), model TM4000Plus (HITACHI, Tokyo, Japan), operated at an accelerating voltage of 10–20 kV. To determine the elemental composition, the SEM was equipped with an energy-dispersive X-ray spectrometer (EDS), model X-stream-2 (Oxford Instruments, Oxford, UK), and data were processed using AZtecOne 1.0 software (Oxford Instruments).

Transmission electron microscopy (TEM) was performed on a FEI Tecnai G^2^ F20 TWIN Cryo-TEM (FEI/Thermo Fisher Scientific, Hillsboro, OR, USA). The analysis encompassed conventional TEM imaging, electron diffraction, and scanning TEM (STEM) modes. Compositional information was obtained via energy-dispersive X-ray spectroscopy (EDX) in STEM mode, utilizing an Oxford Instruments X-Max silicon drift detector (SDD) (Oxford Instruments, Oxford, UK). For sample preparation, the dried powders were dispersed in ethanol and ultrasonicated for 30 min to enhance dispersion. A drop of the resulting suspension was deposited onto a holey carbon grid and allowed to dry, without any staining. This combined approach provided comprehensive structural, morphological, and compositional information about the synthesized materials.

The thermal stability of the samples was evaluated by thermogravimetric analysis (TGA) using a Q5000IR instrument (TA Instruments, New Castle, DE, USA). For each test, 8–10 mg of sample was placed in platinum pans and subjected to a controlled heating program. The temperature was increased at a rate of 10 °C/min up to 750 °C. During heating, high-purity nitrogen (5.0, 99.999%) was used as purge gas 1 at a flow rate of 50 mL/min to provide an inert atmosphere and prevent oxidation. At 750 °C, the purge gas was switched to synthetic air (99.999%) at the same 50 mL/min flow rate, and the temperature was held isothermally for 5 min. The setup allowed monitoring of the samples’ mass changes and decomposition behavior under both inert and oxidative conditions, providing insights into their thermal degradation profiles and stability.

Electrochemical Impedance Spectroscopy (EIS) was used in order to assess the charge resistance transfer at the surface of the modified sensors. The Nyquist plots were recorded for unmodified and modified sensors using a solution of 5 mM of [Fe(CN)_6_]^3−/4−^ prepared in 0.1 M KCl as redox probes, for a frequency range from 1 MHz to 0.1 Hz, at an amplitude of 5 mV imposed at open circuit potential (OCP), for 50 data points. Commercial screen-printed carbon paste electrodes (SPE, 4 mm diameter) on ceramic support from Dropsens were modified with composite mixtures containing the dye-based TiO_2_ samples P3, 3PT and 3PH dispersed in chitosan (CS) matrix. The mixtures were obtained by dispersing 1.5 mg of each sample in 300 µL of 0.5% CS solution, prepared in acetic acid 2%. The obtained mixtures were sonicated for 1 h at room temperature. Volumes of 10 μL were drop-casted on the surface of the working electrode and the modified electrochemical sensors were left overnight at 4 °C.

### 2.4. Preparation of Photocatalytic Coatings

To produce photocatalytic coatings, PH, PT and P-type photocatalysts were blended with the water-based styrene-acrylic paint provided by Chimcolor (Bucharest, Romania) using an Automatic Pigment Muller (J. Engelsmann AG, Ludwigshafen am Rhein, Germany). The resulting coatings were applied to glass slides—pre-cleaned and degreased using Piranha solution—at a thickness of 50 ± 1 μm, using an Elcometer 3570 film applicator (Elcometer Ltd., Manchester, UK). The films were left to dry at room temperature (25 ± 2 °C) for 48 h and were then used for photocatalytic testing. Coating thickness was measured with a TROTEC BB25 layer thickness gauge (TKL GmbH, Heinsberg, Germany).

### 2.5. Evaluation of Coating Photocatalytic Performance

Diffuse reflectance UV–Vis spectra of the powder samples were collected using a JASCO V-570 spectrophotometer (JASCO International Co., Ltd., Tokyo, Japan) fitted with a 150 mm ILN-472 integrating sphere, with Spectralon serving as the reflectance standard. Diffuse reflectance UV-Vis spectra were recorded for coatings deposited on glass slides, using the same setup as employed for photocatalyst measurements. Color evaluations were conducted using the CIE L*a*b* color system with the Jasco VWTS-581 color analysis software (Ver. 2.00A, JASCO Int. Co., Ltd., Tokyo, Japan), using a 10° standard observer and D65 illuminant. Color changes were characterized based on L* which indicates lightness, while a* and b* represent the red-green and yellow-blue components, respectively.

Photocatalytic activity of the coatings was assessed through photodegradation experiments using a volume of 40 mL aqueous solution 6 mg/L of Methylene Blue (MB) as a model pollutant. UV-Vis spectroscopy was used to monitor MB concentration changes during photoreaction.

For detecting the active species during photocatalytic decomposition of model pollutants, the method is similar to the former photocatalytic activity test, using a volume of 40 mL acetonitrile solution 5 × 10^−5^ M of Nitrotetrazolium Blue chloride (NBT). During exposer to light, hydroxyl radicals (•OH) were quenched by adding iPrOH in the ratio sample: iPrOH = 5:1 (by volume).

For comparison, samples were exposed to light in an ATLAS-Xenotest 150S+ chamber (2200 W, Atlas Material Testing Solutions, Mount Prospect, IL, USA), equipped with ATLAS NXe 2000 HE xenon-arc lamp with an IR filter system (spectral range 300–800 nm). The irradiance measured at the level of the tested sample was maintained at 42 ± 2 W/cm^2^. Photodegradation tests in visible light were conducted in a custom-built chamber using OSRAM 200 W LED projectors (OSRAM GmbH, Munich, Germany). The irradiance at the specimen surface was maintained at 30 ± 1 W/m^2^, as quantified with Delta OHM-HD 2302.0 Light-meter (Delta OHM Srl, Padova, Italy) equipped with a calibrated Delta OHM LP471RAD probe, which operates over 400–1050 nm.

## 3. Results

### 3.1. Synthesis of Photocatalytic Composites

In the experimental setup, TiO_2_ was maintained constant at 1 g across all samples, providing a consistent support for dye adsorption. Sensitizers CuPcTC and PTCDA were introduced from 1 mM stock solutions in isopropanol, with masses ranging from approximately 1 mg to 4 mg per sample for single-sensitizer systems, and 2–8 mg for mixed-sensitizer samples (P1–P3). The volumes of the dye solutions and corresponding TBAOH (40% *w*/*w* in water) are summarized in Table 1. Isopropanol volume was kept constant at 20 mL for all samples to ensure uniform dispersion, while TBAOH volume was adjusted proportionally to dye concentration to maintain pH and promote solubilization of the tetrabutylammonium salts. These solutions ensure perfect homogenization with TiO_2_ and an even deposition of the dyes onto its surface. The adjustment of the pH into the neutral range was primarily related to the generation of carboxylic groups capable of anchoring to the TiO_2_ substrate and preventing dissolution of the dyes in the aqueous media in which the photocatalytic degradation of model pollutants (e.g., methylene blue) is performed. Moreover, the protonated Ti–O–O–Ti intermediate at near-neutral pH facilitates efficient transfer of photogenerated holes, thereby promoting the formation of hydroxyl radicals (•OH). Under alkaline conditions (pH ≥ 9.6), deprotonation of this surface intermediate diminishes the density of active peroxo-like species capable of hole trapping. Consequently, interaction with photogenerated holes becomes less effective, inhibiting subsequent oxidation steps and sharply reducing •OH generation efficiency to approximately 0.01–0.05%, compared to 0.2–0.6% at near-neutral pH [42,43].

### 3.2. Energetic Diagram and Photocatalytic Generation of Reactive Species

**Figure 1:**
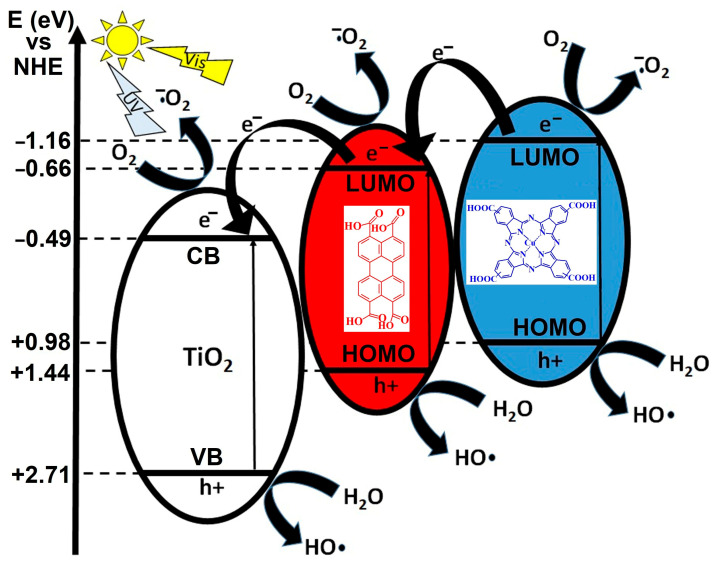
Energy-level alignment diagram of the heterojunction components [44,45,46].

Figure 1 illustrates a proposed photocatalytic mechanism involving a ternary heterojunction system composed of TiO_2_ (left), a red-colored organic semiconductor (center, PTCA), and a blue-colored phthalocyanine-based compound (right). The energy diagram is referenced to the normal hydrogen electrode (NHE), with conduction band (CB) and valence band (VB) positions marked in electron volts (eV). Under visible light (hν_vis) irradiation, both the organic semiconductors and TiO_2_ can be photoexcited, generating electron–hole (e^−^/h^+^) pairs. The diagram shows an electron transfer cascade in which photogenerated electrons from the red organic semiconductor’s CB are transferred to the CB of TiO_2_, while electrons from the blue CuPcTC CB can also migrate towards oxygen reduction pathways. This stepwise electron migration minimizes recombination losses and facilitates effective charge separation.

The CB potentials of the red and blue components are more negative than the O_2_/•O_2_^−^ reduction potential (−0.16 eV), enabling the photogenerated electrons to reduce molecular oxygen to superoxide radicals (•O_2_^−^). These reactive oxygen species are highly oxidative and play a critical role in pollutant degradation and antimicrobial activity. The diagram depicts multiple points where O_2_ is reduced to •O_2_^−^, suggesting parallel electron pathways from different CB levels. In parallel, the photogenerated holes in the VB of all three components are sufficiently positive to oxidize water (H_2_O) or hydroxide ions (OH^−^) to hydroxyl radicals (•OH), which are among the most powerful oxidants in advanced oxidation processes. The VB of TiO_2_ (+2.71 eV) and the red and blue components are all located above the •OH/H_2_O oxidation potential, ensuring this reaction is thermodynamically feasible.

This arrangement of energy levels indicates a Z-scheme or stepwise heterojunction mechanism, where photogenerated electrons and holes are spatially separated across the materials, enhancing redox efficiency. The semiconducting chromophores act as visible light sensitizers, extending the light absorption range beyond TiO_2_’s UV-active domain, thereby increasing solar energy utilization. The sequential charge transfers and cooperative radical generation pathways are designed to maximize photocatalytic efficiency, reduce electron–hole recombination, and produce a high yield of reactive oxygen species for pollutant degradation or sterilization applications.

The figure represents a synergistic photocatalytic system where TiO_2_ provides structural and oxidative stability, PTCA ensures strong visible light absorption and electron donation, and CuPcTC facilitates broad-spectrum light harvesting and efficient oxygen reduction. This combination results in enhanced photocatalytic activity under visible light through efficient charge separation, expanded absorption range, and possibility of simultaneous generation of •OH and •O_2_^−^ radicals.

### 3.3. UV-Vis Diffuse Reflectance Spectroscopy (DRS)

**Figure 2:**
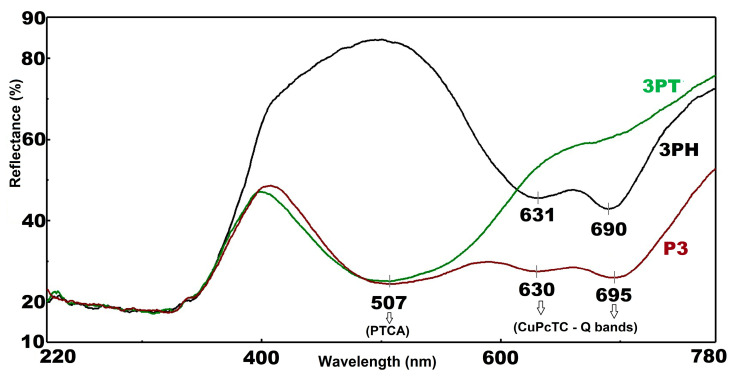
UV-Vis Reflectance spectra of hybrid photocatalysts.

In Figure 2 all three samples exhibit low reflectance (~20%), characteristic of strong UV absorption due to fundamental band-to-band transitions in semiconductor photocatalysts. This behavior aligns with typical UV absorption edges in materials such as TiO_2_. A pronounced increase in reflectance occurs in this region, reaching a mid-range peak (~45–50%)—indicative of a spectral window where the photocatalysts reflect rather than absorb, likely tied to their color-inducing electronic transitions.

The three sensitized TiO_2_ materials exhibit well-resolved and chemically distinct optical signatures—3PT displays exclusively the characteristic PTCA absorption in the 450–550 nm range, while 3PH exhibits only the CuPcTC Q-band features in the 600–700 nm region; by contrast, P3 co-sensitized material clearly manifests both dye absorptions, confirming that both species successfully adsorb and retain their characteristic spectral fingerprints. Furthermore, no significant shifts are observed in any of the peak positions relative to the free, monomeric dye maxima, indicating moderate surface binding to TiO_2_ with minimal molecular aggregation. The relative intensities of the reflectance minima correspond to dye loading—in P3 the PTCA minimum at about 507 nm is as deep as in the single-dye 3PT sample, indicating effective co-loading, whereas the CuPcTC Q-band in P3 is slightly attenuated relative to 3PH—possibly due to competitive surface adsorption or altered packing. Bands are very well evidenced in Figure S1 which show the Kubelka-Munk conversion of the reflectance spectra. A trough in reflectance—strongest for sample P3—suggests absorption features such as d–d transitions or charge transfers. This aligns with known behavior in transition metal–doped oxides, where reflectance minima signal specific electronic absorption bands. The 3PT shows a steep rise in reflectance starting near 630 nm, with 3PH following at around the same point. These upward trends imply reduced absorption or onset of NIR reflectance in these materials. Another dip appears around this wavelength, most notable in 3PH and P3, possibly due to overlapping weak electronic transitions or vibrational overtone features. Similar absorption features in NIR have been tied to overtones or d–d transitions in metal-doped materials, such as CuPcTC.

P3, with its deeper troughs and lower overall NIR reflectance, may exhibit stronger light absorption—potentially enhancing photon capture but increasing heat generation. On the other side, 3PT, with its elevated NIR reflectance, is promising for solar-reflective or thermally efficient coatings (“cool” materials), while 3PH falls intermediate, suggesting a balance of absorption and reflectance tailored for specific photocatalytic or optical roles.

The Figure S2 presents the Tauc plot analysis of PTCA sensitized P25 (3PT), CuPcTC sensitized P25 (3PH), and PTCA/CuPcTC dual sensitized P25 (P3). The absorption edges in the high-energy region (around 3.08–3.17 eV) correspond to the intrinsic band gap of TiO_2_ (P25), which remains relatively consistent across the three systems with slight variations depending on the sensitizer employed. Specifically, the estimated optical band gaps are 3.08 eV for the dual-sensitized P3, 3.13 eV for 3PT, and 3.17 eV for 3PH. These small differences suggest that the introduction of the organic sensitizers slightly modifies the electronic interactions at the semiconductor interface, but the fundamental TiO_2_ band edge absorption is largely preserved.

In the lower-energy region (1.6–1.92 eV), the sensitizers contribute additional optical transitions, highlighting their role in extending light absorption into the visible range. For 3PT, a sub-band gap transition appears at approximately 1.92 eV, while 3PH exhibits an absorption onset near 1.65 eV. The dual-sensitized P3 system shows distinct states at 1.6 eV and 1.65 eV, reflecting the combined contributions of both PTCA and CuPcTC. These sub-band gap features arise from sensitizer-derived states and defect-level interactions, which facilitate improved visible light harvesting. Thus, the comparison illustrates how single and dual sensitization strategies tune the optical response of P25, enhancing its suitability for light-driven applications such as photocatalysis and solar energy conversion.

### 3.4. DRIFT Spectroscopy

**Figure 3:**
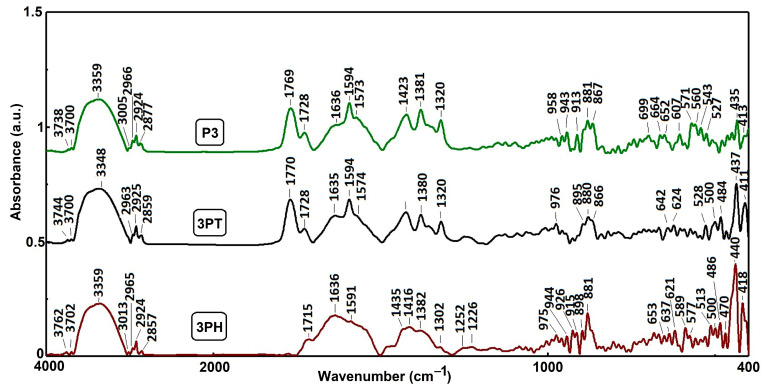
DRIFT spectra of photocatalytic samples P3, 3PT, 3PH.

In Figure 3 all three samples display broad absorption bands between 3740–3760 cm^−1^ down to ~3348 cm^−1^. These are commonly attributed to O–H stretching, indicating surface hydroxyl groups or adsorbed moisture on TiO_2_ surfaces. The slight shifts among samples reflect interactions or binding variations between TiO_2_, CuPcTC, PTCA.

Distinct peaks near 3000, 2960, 2920, and 2850 cm^−1^ likely correspond to C–H stretching vibrations. These are expected from the organic moieties in both PTCA and CuPcTC and also in TBAOH used for solubilization, indicative of alkyl groups or aromatic ring environments in these compounds. Strong peaks observed in the 1728–1770 cm^−1^ region may be linked to C=O stretching of carboxylic acids and anhydride at higher values of the wavenumber. The band corresponding to C=O stretching of anhydride group appears in the analyzed samples as a result of the drying process at 125 °C, due to the presence of adjacent free carboxylic groups in PTCA, as evidenced in Figure S3.

The DRIFT spectrum of sample P3 (Figure S4) reveals the distinct vibrational modes associated with carboxyl functional groups. The absorption at 1725 cm^−1^ (A = 0.88) corresponds to the stretching vibration of protonated carboxyl groups (νC=O), while the more intense peaks at 1594 cm^−1^ (A = 1.14) and 1381 cm^−1^ (A = 1.27) are attributed to the asymmetric (ν_as_ COO^−^) and symmetric (ν_sym_ COO^−^) stretching vibrations of deprotonated carboxylates, respectively. Such assignments are in line with standard IR data for polycarboxylic aromatic acids and derivatives.

The efficiency (η) of carboxylate binding on TiO_2_, calculated as the ratio of the carboxylate band intensities relative to the C=O band of protonated carboxyl groups, is:

(1) $$\eta =\frac{A_{asym{COO}^{-}}+A_{sym{COO}^{-}}}{A_{C=O}}$$

The high efficiency (η ≈ 2.74) observed in P3 suggests that most of the carboxyl groups exist in the deprotonated –COO^−^ state, which facilitates strong coordination with the TiO_2_ surface. If we extend the efficiency (η) of carboxylate binding on TiO_2_ as a descriptor of multidentate coordination potential, a value of >2.5 indicates that most carboxyl groups can participate simultaneously in surface binding, maximizing surface contact points. This implies that in P3, organic molecules are likely anchored to TiO_2_ predominantly through multidentate carboxylate linkages, which are known to increase stability against desorption, improve electronic coupling, and facilitate charge transfer between the organic chromophore and the inorganic semiconductor substrate [47].

Peaks between 1636 and 1594 cm^−1^ are likely associated with C=C or C=N stretching in aromatic or conjugated ring systems. These are characteristic of both PTCA’s conjugated architecture and the phthalocyanine macrocycle present in CuPcTC.

Multiple bands between 1423–1320 cm^−1^ can be assigned to C–H bending, possibly aromatic ring vibrations or conjugated system modes in the organic compounds.

In the low-wavenumber region (≈980–400 cm^−1^), notable peaks such as those around 976, 943, 881, 867 cm^−1^ (3PT and 3PH) and 975–915, 881 (P3) are present, which may correspond in part to Ti–O–Ti stretching or bending vibrations, generally seen below 700 cm^−1^ in TiO_2_ spectra. Additionally, small, sharp features between 650–470 cm^−1^ likely align with the characteristic anatase Ti–O–Ti modes.

The FTIR spectra clearly differentiate the three sample compositions: 3PH showcases strong aromatic and phthalocyanine-specific signatures; 3PT highlights carbonyl-rich, conjugated molecular features specific to PTCA; P3 combines vibrational signatures of both organic components alongside TiO_2_-related modes.

### 3.5. XRD Analysis

**Figure 4:**
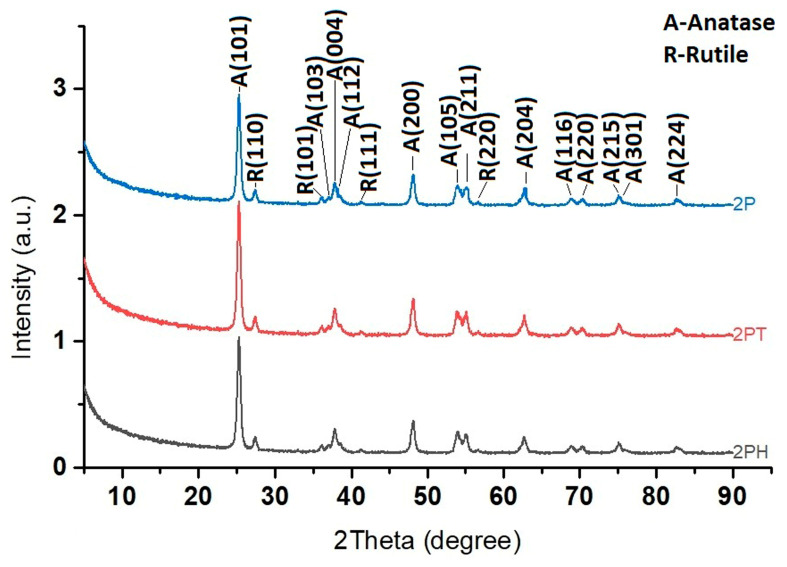
XRD pattern of the hybrid photocatalysts (P2; 2PT; 2PH).

In Figure 4, two crystalline phases are identified: Anatase (A) and Rutile (R), both polymorphs of titanium dioxide (TiO_2_). Each peak is indexed with the Miller indices (hkl) of the corresponding crystal planes. The annotation “A-Anatase” and “R-Rutile” clarifies the phase assignments. For example, A(101) at around 25° corresponds to the anatase phase, while R(110) at around 27° is a signature peak for rutile.

From the patterns, several observations can be made: The 2P sample exhibits multiple peaks corresponding to both anatase and rutile phases, suggesting it is a mixed-phase TiO_2_. The anatase peaks dominate, but noticeable rutile peaks (e.g., R(110), R(101), R(111), and R(220)) are also present. In the case of 2PT sample, the diffraction peaks are still from TiO_2_, but the anatase peaks appear more pronounced relative to rutile. The pattern of 2PH shows sharper and fewer peaks, indicating higher crystallinity but possibly a single dominant phase. The major peak at ~25° (A(101)) suggests predominantly anatase TiO_2_, with little rutile phase.

The XRD patterns of TiO_2_ samples, before and after deposition of dyes, display the characteristic reflections of anatase and rutile without any extra crystalline phases, confirming that the bulk crystal structure remains unaltered. Nevertheless, systematic variations in the relative intensities of the anatase (101), (001) and rutile (110) reflections are evident. These variations, rather than indicating phase transformation, reflect changes in preferred orientation and exposed facet distribution, which can be modulated by the facet-selective adsorption of dye molecules onto specific crystal planes. Such surface-induced morphological adjustments can subtly alter the textural XRD signature even in the absence of structural re-ordering.

Facet selectivity is a key factor behind these physical signatures. PTCA, through its multidentate carboxyl groups, shows strong preferential binding to rutile facets—especially the (110) plane—which offers a periodic array of five-fold coordinated Ti atoms supportive of bidentate anchoring [48]. In contrast, anatase (101) typically presents fewer coordination sites and thus lower binding affinity; significant adsorption is observed only on high-energy planes like (001) which are less exposed under standard synthesis. CuPcTC, with a larger π-conjugated macrocycle and four carboxyl anchor groups, can engage in even more robust multidentate interactions across both polymorphs; their strong adsorption potentially stabilizes rutile facets more effectively within mixed-phase systems. Recent work [49] shows that rutile (111) facets display markedly higher photocatalytic activity—even in the absence of Pt co-catalyst—while low-energy facets like rutile (110) require Pt loading to become effective. This facet-specific reactivity reflects underlying differences in surface energy, band edge positions, and molecular binding strength.

These insights give new meaning to the XRD data: although organic dyes are invisible in diffraction, their binding preferences influence the relative reflection intensities, peak broadening, and apparent anatase–rutile ratios. As such, changes in XRD peak intensity serve as an indirect yet powerful probe of interfacial chemistry and becomes part of an integrated toolkit for understanding how facet-selective dye adsorption influences morphology, surface exposure, and ultimately the photocatalytic efficiency of the TiO_2_–dye systems.

### 3.6. TEM and Elemental Composition by EDX

**Figure 5:**
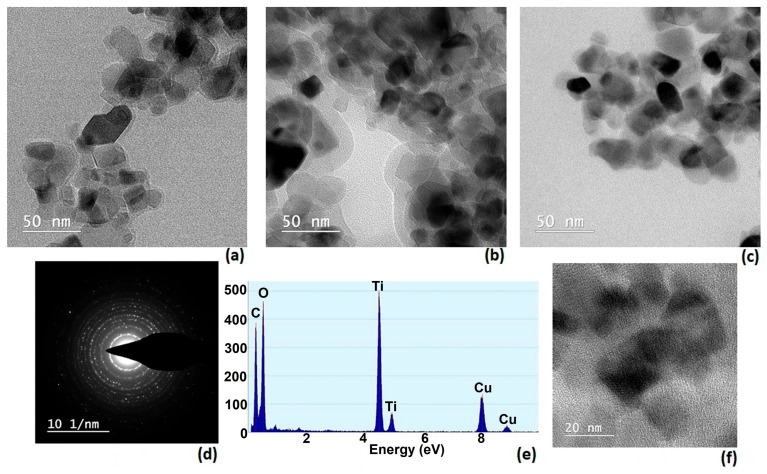
TEM images of 3PT (**a**), 3PH (**b**), P3 (**c**); P3 SAED (**d**); P3 EDX (**e**); P3 TEM (**f**).

In Figure 5, a network of dark regions represents the projected shadows of individual particles or clusters of particles, while the lighter background corresponds to the electron-transparent support film. The contrast arises from differences in electron scattering, which depend on the atomic composition and thickness of the observed regions. Particles in the darker areas are typically denser or thicker, while faint outlines indicate thinner or more electron-transparent zones.

The observed morphology suggests that the material consists of nanoscale particles with irregular but relatively uniform shapes. Some particles appear loosely aggregated, forming small clusters, while others remain isolated. This type of arrangement is often observed in materials prepared by co-precipitation, sol–gel, or hydrothermal synthesis, where nanoparticles tend to agglomerate due to high surface energy. The uniformity of particle size across the image hints at a controlled synthesis process, which is important for ensuring reproducible material properties.

TEM analysis is crucial for correlating structure with function. In catalytic materials, smaller and more uniform particles generally offer higher surface area and improved activity, as in this case.

In the presented SAED image (Figure 5d), distinct diffraction spots arranged in ring-like formations can be observed, indicating that the sample contains multiple crystalline domains oriented in different directions—typical of polycrystalline materials. Each ring corresponds to a specific set of crystallographic planes, and the radii of these rings are related to the interplanar spacing according to Bragg’s law. The sharpness and uniformity of the rings suggest good crystallinity, while the number of visible rings provides information about the range of lattice spacing present in the sample. This type of analysis is often combined with XRD data to confirm phase composition and lattice parameters at a higher spatial resolution.

EDX analysis confirms the presence of titanium and oxygen belonging to the inorganic photocatalyst and copper associated with the presence of CuPcTC dyestuff in P3 sample. In Figure 5e carbon and oxygen suggest the presence of organic species at the surface of TiO_2_.

### 3.7. SEM-EDS Analysis

**Figure 6:**
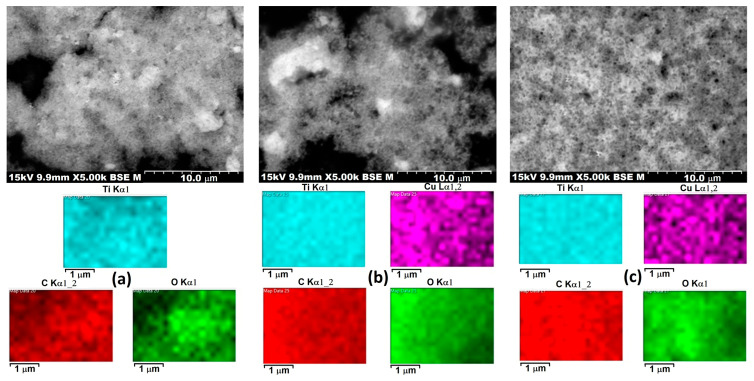
SEM and EDS images of (**a**) 3PT; (**b**) 3PH; (**c**) P3.

Figure 6 presents scanning electron microscopy (SEM) images combined with energy-dispersive X-ray spectroscopy (EDS) elemental mapping for three different samples labeled 3PT, 3PH, and P3. The top row shows SEM micrographs at 5000× magnification, revealing surface morphology and particle distribution, while the lower panels display the corresponding elemental maps for titanium (Ti, cyan), copper (Cu, magenta), carbon (C, red), and oxygen (O, green).

In terms of morphology, all three samples exhibit an agglomerated nanoparticle network with irregular shapes, typical of TiO_2_-based composite materials. The particle clusters appear to have submicron to micron-scale agglomerates, with 3PT and 3PH showing more prominent bright regions in the backscattered electron images, which often correspond to higher atomic number elements such as Ti or Cu. P3 displays a more uniform grayscale distribution, suggesting a more homogeneous material or smaller particle size distribution without pronounced high-density regions.

The EDS elemental mapping reveals that titanium and oxygen are evenly distributed throughout all samples, confirming the presence of a TiO_2_ matrix as the main structural component. Carbon mapping shows homogeneous coverage, which could be attributed to organic ligands, carbon-based modifiers, or surface adsorbates. Copper distribution, visible in 3PH and P3, appears as evenly dispersed magenta signals, indicating successful incorporation or deposition of Cu species without large-scale clustering. This uniform dispersion is beneficial for catalytic or photocatalytic activity since it maximizes active site availability.

A notable difference between the samples lies in the apparent density and distribution of copper. In 3PH, Cu appears slightly more intense in localized regions, which may indicate areas of higher surface concentration or partial aggregation. In contrast, P3 shows a more diffuse and uniform Cu distribution, which might enhance stability and interaction with the TiO_2_ surface. 3PT’s Cu map is absent, suggesting either a lower copper loading or a different compositional modification.

The SEM and EDS results demonstrate that all three materials have a nanoscale agglomerated morphology with well-distributed Ti, O, and C, while the presence and dispersion of Cu vary between 3PH and P3 samples. These differences in microstructure and elemental dispersion could influence the materials’ surface reactivity, photocatalytic efficiency, and durability in practical applications. The high uniformity of elemental distribution, especially for Cu in P3 and 3PH, is indicative of effective synthesis and strong potential for catalytic applications.

### 3.8. Thermogravimetric Analysis

**Figure 7:**
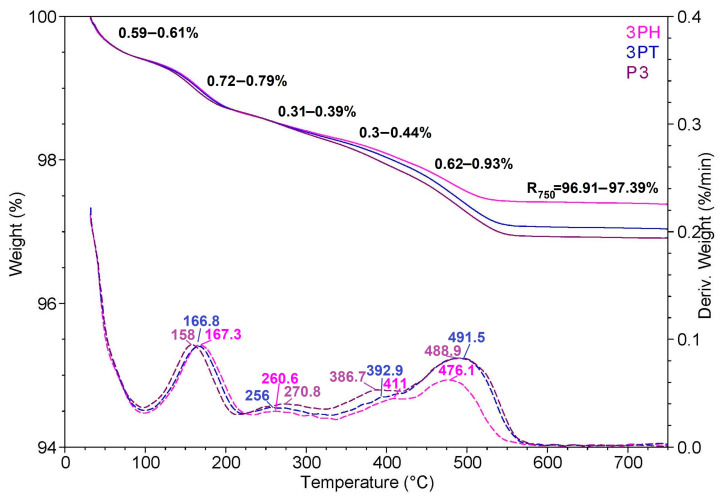
Thermogravimetric analysis of hybrid photocatalysts.

Figure 7 shows thermogravimetric analysis (TGA) and derivative thermogravimetric (DTG) curves for three samples labeled 3PH, 3PT, and P3. The weight loss curves (solid lines) are plotted against temperature on the left axis, while the DTG profiles (dashed lines) are plotted on the right axis as the rate of weight loss (%/min).

All three samples display similar thermal stability profiles, with only minor variations in the onset and extent of degradation. The first slight weight loss occurs below ~150 °C, which can be attributed to the removal of adsorbed or bound water and possibly small volatile molecules. This stage is accompanied by a small DTG peak, indicating a low-rate mass loss process.

A second noticeable weight loss step appears between ~150 °C and 250 °C, corresponding to the degradation of surface functional groups or the decomposition of low-molecular-weight organic residues. The DTG curves in this range show a small but distinct peak for all samples, confirming a common decomposition mechanism.

The major weight loss event is observed between ~450 °C and 550 °C, where the DTG curves exhibit their highest peaks. This stage likely corresponds to the thermal decomposition of the main organic framework or ligand components present in the materials. After this event, the weight stabilizes, indicating that the remaining material is composed mainly of thermally stable inorganic components such as metal oxides.

Although the overall thermal stability trend is similar for 3PH, 3PT, and P3, subtle differences are visible: 3PH retains slightly more weight at higher temperatures, suggesting marginally better thermal resistance, while P3 shows a slightly higher decomposition rate during the main degradation step. These variations may be linked to differences in composition, surface modification, or structural packing.

Overall, the TGA/DTG profiles indicate that all three materials are stable up to about 400 °C, with main decomposition occurring in a high-temperature range, confirming their potential suitability for applications requiring moderate to high thermal resistance.

### 3.9. Electrochemical Impedance Spectroscopy

The Figure S5 displays the Nyquist plots obtained from electrochemical impedance spectroscopy (EIS) measurements for different systems: SPE, CS/SPE, CS-P3/SPE, CS-3PT/SPE, and CS-3PH/SPE. The main plot shows the imaginary component of impedance as a function of the real component in the kilohm range, while the inset provides a magnified view in the lower resistance region (100–180 Ω) for better comparison. The bare SPE electrode exhibits a significantly larger semicircle, indicating higher charge-transfer resistance and poor interfacial conductivity. By contrast, all composite-modified electrodes (CS/SPE, CS-P3/SPE, CS-3PT/SPE, and CS-3PH/SPE) demonstrate a notable decrease in impedance, highlighting the beneficial role of the sensitizer-modified TiO_2_ materials in enhancing interfacial charge transport.

Among the modified electrodes, the inset reveals differences in the extent of impedance reduction depending on the sensitization strategy. The CS-3PT/SPE (Rct = 27.39 Ω) and CS-P3/SPE (Rct = 26.60 Ω) electrodes exhibit the lowest charge-transfer resistance, as evidenced by their smaller semicircles compared to CS-3PH/SPE (Rct = 28.87 Ω) and CS/SPE (Rct = 54.53 Ω). This improvement suggests more efficient electron transfer dynamics and stronger interfacial coupling between the sensitized materials and the electrode. The performance variation can be attributed to the synergistic effects of PTCA and CuPcTC sensitizers, with dual sensitization (CS-P3/SPE) achieving balanced charge-transfer properties. These results confirm that functionalization with organic sensitizers effectively reduces interfacial resistance, thereby improving the overall electrochemical performance of the system.

The bare SPE electrode shows a very large Rct (1882.6 Ω), indicating poor interfacial charge transfer compared with any of the composite-modified electrodes. All sensitized/CS-modified electrodes reduce Rct by roughly an order of magnitude, with CS-3PT/SPE and CS-P3/SPE appearing to give the lowest interfacial resistance (best charge transfer) in this dataset. The differences between the modified electrodes are modest but consistent with improved electronic coupling/charge extraction when PTCA and/or CuPcTC are present; the dual-sensitized sample (CS-P3) and the PTCA-sensitized sample (CS-3PT) show particularly low Rct, which would be expected to correlate with improved electrochemical (e.g., photocatalytic or sensor) performance.

## 4. Discussion

**Figure 8:**
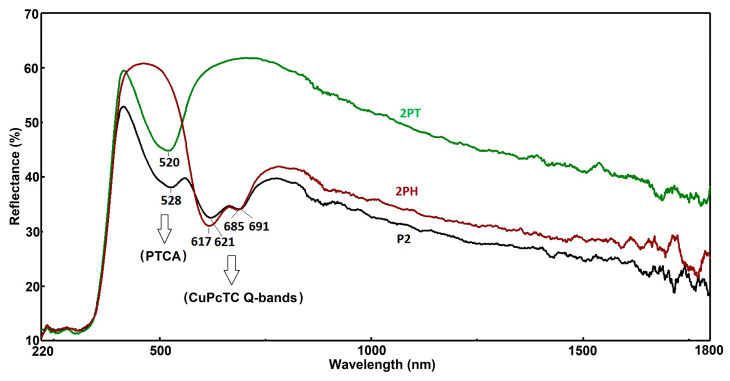
UV-Vis-NIR diffuse reflectance spectra of the coatings containing the three types of photocatalysts.

In the study of the photocatalytic properties used to select the most effective materials, the evaluation was conducted using photocatalytic coatings deposited on glass slides, which were immersed in the test solutions. The UV–Vis–NIR reflectance spectra of the photocatalytic coatings are shown in Figure 8.

Figure 8 presents diffuse reflectance spectra (%R) for three different samples—labeled P2, 2PH, and 2PT—across the wavelength range of 220–1800 nm. These spectra provide insight into the optical properties, light absorption behavior, and possible surface modifications of the materials. In the visible range, distinct differences are observed in the reflectance profiles, particularly in the spectral regions around 520–530 nm and 617–691 nm, which correspond to key electronic transitions likely associated with pigment or semiconductor absorption features. The P2 sample (black curve) displays the lowest reflectance throughout most of the spectrum, indicating higher light absorption capacity, which could be beneficial for photocatalytic or photothermal applications. In contrast, the 2PT sample (green curve) exhibits the highest reflectance across the spectrum, especially in the near-infrared region (>800 nm), suggesting reduced light absorption and potentially different surface morphology or composition that enhances reflectivity. The 2PH sample (red curve) lies between P2 and 2PT in reflectance behavior, showing moderate absorption with slightly higher reflectance than P2 but lower than 2PT.

Notable features include the strong reflectance peaks near 520 nm for 2PT and 528 nm for P2, as well as at 528 nm and 520 nm for 2PH, which may indicate slight shifts in optical band gap absorption edges due to compositional or structural variations. Similarly, the peaks and shoulders at 617–621 nm and 685–691 nm suggest the presence of specific chromophoric species or transitions that are retained in all samples but with varying intensities. These differences could be due to doping, surface functionalization, or post-treatment processes affecting the electronic states and scattering properties.

In the near-infrared (NIR) region, 2PT maintains the highest reflectance, implying a lower degree of free carrier absorption or reduced defect-related absorption compared to P2 and 2PH. This characteristic might make 2PT more suitable for applications requiring thermal management or minimized solar heat gain. On the other hand, the strong light absorption of P2 in both the visible and NIR ranges suggests it would be more effective in photothermal conversion or photocatalytic processes where maximum photon harvesting is desired. The intermediate behavior of 2PH suggests a balance between absorption and reflection, potentially offering a compromise between optical efficiency and thermal control.

Overall, the reflectance spectra reveal that surface modification or compositional changes between P2, 2PH, and 2PT have a significant influence on light–matter interaction. The red shift or blue shift of characteristic peaks, combined with differences in baseline reflectance, points to alterations in electronic structure and scattering mechanisms. Such differences could be strategically exploited depending on the desired application—whether it is maximizing light absorption for energy conversion or optimizing reflectance for thermal management.

Hydroxyl radicals (•OH)—either surface-bound or free in solution—play a critical downstream role, particularly when direct hole transfer is inhibited by surface modifiers, competitive adsorption, or the presence of radical scavengers. The balance between direct-hole vs. hydroxyl-radical mechanisms is strongly influenced by TiO_2_ parameters—the crystal phase (anatase vs. rutile vs. brookite), surface chemistry (e.g., presence of modifiers, site blocking), pollutant adsorption affinity, and the presence of radical scavengers or promoters in the reaction matrix. All of these factors modulate hole availability and radical generation, shifting the dominant oxidation pathway depending on the specific system conditions [50].

When TiO_2_-based systems are sensitized with PTCA or CuPcTC, visible light absorption is enhanced and electron injection into TiO_2_ occurs, generating photogenerated holes (h^+^) on the TiO_2_ valence band. However, across both undoped and sensitized TiO_2_ systems, numerous studies consistently show that indirect ·OH-mediated radical pathways dominate the degradation of organic pollutants, rather than direct oxidation by surface holes. Indeed, comparative analyses between TiO_2_ and other semiconductors (e.g., BiOCl) indicate that TiO_2_ preferentially oxidizes water or surface hydroxyl groups to produce hydroxyl radicals (·OH), which then diffusively attack organics, rather than transferring holes directly to the pollutant molecule [51].

Specifically, for CuPcTC- or PTCA-sensitized TiO_2_, visible light activation promotes charge separation such that injected electrons reduce O_2_ to superoxide (·O_2_^−^), and the corresponding holes oxidize water or surface hydroxyls to ·OH. Reactive-species scavenging experiments typically show strong inhibition of degradation upon addition of ·OH scavengers like tert-butanol or p-benzoquinone, pointing to radical-driven mechanisms. For example, CuPcTC–TiO_2_ systems have demonstrated significantly enhanced degradation performance under visible light, yet the dominant active species remain hydroxyl (·OH) and superoxide radicals, not direct hole transfer to organics [52].

**Figure 9:**
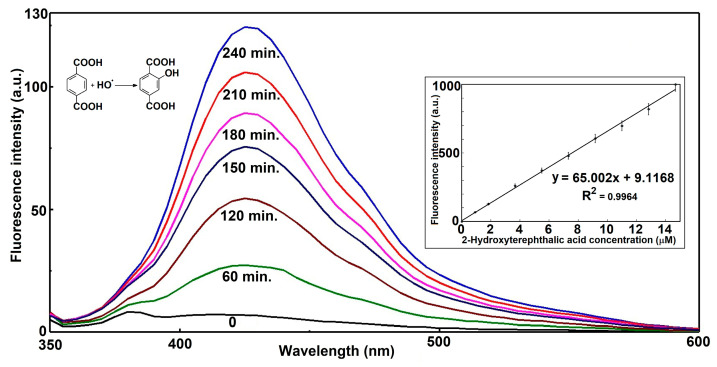
Estimation of photochemical generated hydroxyl radicals at the surface of P3 coatings under arc-xenon light illumination by formation of 2-hydroxyterephthalic acid (inset—calibration curve of HTPA).

The presented fluorescence emission spectra in Figure 9 depict the time-dependent formation of hydroxyterephthalic acid (HTPA) from the hydroxylation of terephthalic acid (TPA) by hydroxyl radicals (•OH). Across all measured time intervals, the emission maxima remain centered around approximately 425 nm, confirming that a single fluorescent species—HTPA—is responsible for the signal. At the initial time point (0 h), the spectrum is almost flat, indicating negligible fluorescence and confirming that no significant product had formed prior to radical exposure. As the reaction proceeds, the fluorescence intensity steadily increases, with notable growth from 1 h to 4 h, where the peak intensity surpasses 120 a.u. This continuous rise in signal intensity is consistent with progressive accumulation of HTPA as hydroxyl radicals react with TPA.

The inset plot further supports the linearity between fluorescence intensity and molar concentration of HTPA in a relatively large range. Importantly, the •OH generation rate is sustained throughout the 4 h measurement period, without signs of depletion of reactants or catalyst deactivation. This observation implies a stable and efficient radical generation process, likely under constant environmental and experimental parameters.

Mechanistically, the linear relationship between fluorescence intensity and time indicates that the concentration of generated •OH is directly proportional to irradiation or reaction duration. This is a key characteristic in systems such as photocatalytic •OH generation or Fenton-type reactions operating under steady-state radical production. Moreover, the stability of the emission wavelength implies that no significant side reactions or secondary fluorescent products interfere with the detection, ensuring reliability of HTPA as a probe molecule.

In summary, the data provide strong evidence for efficient, constant-rate hydroxyl radical generation over the four-hour experimental window, leading to proportional and measurable accumulation of the fluorescent hydroxylation product. The combination of a steady increase in fluorescence intensity, a consistent spectral profile, and a high-quality linear correlation fit underscores both the robustness of the reaction process and the suitability of TPA fluorescence as a monitoring tool for •OH detection in this system.

**Figure 10:**
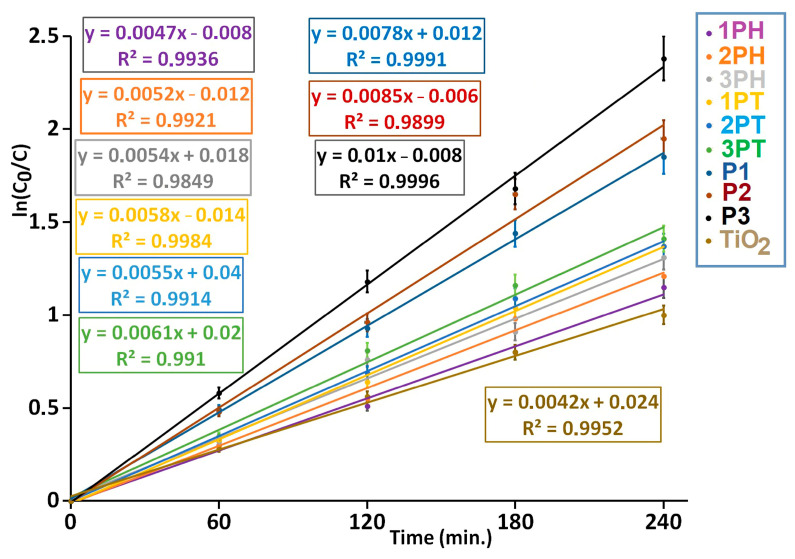
Evolution of the photocatalytic degradation of MB at the surface of coatings containing 10% by weight photocatalysts.

In our experiments, the photocatalytic degradation of MB under a xenon-arc lamp follows a pseudo-first-order kinetics model, evidenced by excellent linear fits (R^2^ ≈ 0.99 or higher). The apparent rate constants k′ obtained from the slopes of ln(C_0_/C) vs. time are summarized in Figure 10. Among all samples, P3 shows the highest activity (k′ ≈ 0.010 min^−1^, t_1/2_ ≈ 69 min), followed by P2 and P1, whereas TiO_2_ (pristine) exhibits the slowest kinetics (k′ ≈ 0.0042 min^−1^, t_1/2_ ≈ 165 min.). These results underscore that modified or composite catalysts outperform bare TiO_2_ under visible-rich irradiation sources. Literature reports of MB degradation using TiO_2_ under UV/visible irradiation often yield pseudo-first-order rate constants in the range from ~0.0035 to ~0.018 min^−1^ depending on catalyst modification, deposition, or composite formation [53,54].

In conclusion, under xenon-arc light (serving as a solar-spectrum simulator), catalysts P3 > P2 > P1 outperform the unmodified TiO_2_ by a factor of ~2–2.5 in terms of apparent rate constants. This aligns with studies reporting that doped or heterostructured TiO_2_ systems enhance visible light reactivity and suppress charge recombination, leading to faster pseudo-first-order degradation kinetics [55,56]. These findings justify that samples are genuinely more efficient in MB photocatalytic decomposition under broad-spectrum irradiation. In Figure S6 is presented the photocatalytic degradation of MB at the surface of P3 coating under xenon-arc illumination.

Mechanistically, the photocatalytic degradation likely involves the generation of electron–hole pairs upon light absorption by the photocatalyst. Photogenerated holes (h^+^) can directly oxidize MB or react with water/hydroxide ions to form hydroxyl radicals (•OH), while photogenerated electrons (e^−^) reduce dissolved oxygen to superoxide radicals (O_2_•^−^). These reactive oxygen species then attack the MB molecules, breaking down the conjugated aromatic structure and leading to loss of the characteristic absorption band. The progressive decline in absorbance with reaction time reflects the cumulative effect of these oxidative processes.

Overall, the data clearly demonstrate the high efficiency of the photocatalytic system in degrading MB, with significant color removal achieved within the 4 h experimental window. The strong correlation between reaction time and absorbance decline confirms a stable and sustained degradation rate, underscoring the photocatalyst’s potential for wastewater treatment applications targeting dye pollutants. The absence of new absorption peaks further suggests that intermediate colored products are either not formed in detectable quantities or are rapidly mineralized into non-absorbing species such as CO_2_ and H_2_O.

The UV-Vis absorption spectra presented in Figure S7 provide evidence for the formation of reactive oxygen species (ROS), specifically superoxide anion radicals (O_2_•^−^), during the photocatalytic process involving the material labeled P3. Nitro blue tetrazolium (NBT), a well-known probe for detecting superoxide radicals, was used to assess ROS generation under various conditions. In the presence of P3 alone (NBT/P3), there is a week to moderate increase in absorbance in the 500–600 nm region, which corresponds to the formation of formazan, the reduced product of NBT upon reaction with superoxide radicals. This indicates that the photocatalyst P3 can generate O_2_•^−^ under the given experimental conditions. The absorbance increase becomes more pronounced when hydrogen peroxide (H_2_O_2_) is added to the system (NBT/H_2_O_2_/P3), suggesting an enhanced generation of superoxide radicals, likely through photocatalytic decomposition of H_2_O_2_ facilitated by P3.

The inset graph further highlights the relative absorbance differences among the three systems (NBT, NBT/P3, and NBT/H_2_O_2_/P3), clearly demonstrating a progressive enhancement in ROS formation. The significant increase in absorbance for the NBT/H_2_O_2_/P3 system suggests that the interaction between H_2_O_2_ and the photocatalyst promotes additional ROS production, potentially via a photo-Fenton-like mechanism. This observation supports the role of P3 in activating molecular oxygen or H_2_O_2_ to generate superoxide radicals upon light irradiation, which are critical intermediates in oxidative photocatalytic degradation pathways. These findings affirm the capability of P3 to facilitate photocatalytic reactions involving ROS and provide insight into the mechanism underlying its activity.

**Figure 11:**
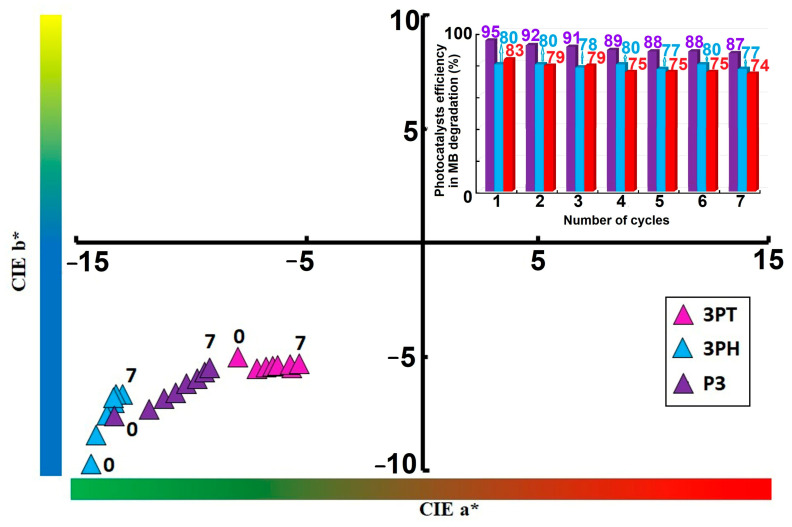
Trajectories of CIE color parameters (a* vs. b*) over seven photocatalytic cycles for coatings 3PT, 3PH, and P3 (inset—photocatalytic efficiency in MB degradation during 7 cycles).

Tracking CIE a*/b* values can serve as a non-destructive metric for assessing photocatalyst degradation or surface aging over operational cycles. All samples migrate toward increasing a*and b* values—shifting diagonally upward and rightward, as it can be seen in Figure 11. This suggests the coatings exhibit a perceptible shift toward redder and yellower tones after repeated use.

3PT displays the largest positive shift in both a* and b* coordinates, indicating a more marked color change. P3 experiences an intermediate shift, showcasing its moderate but noticeable alteration over seven cycles. 3PH exhibits the smallest migration, implying better color stability across photocatalytic cycles. Values shifting toward positive a* imply the coatings develop more reddish tones with repeated photocatalysis—potentially due to accumulation of intermediate species or partial surface modifications. Movement toward positive *b* indicates progression toward warmer, yellowish hues. This may reflect photogenerated compounds or subtle compositional changes influencing how the surface scatters or absorbs visible light.

The color shifts—especially pronounced in 3PT—suggest material changes (e.g., surface oxidation, dopant state changes) that may affect both aesthetic and functional longevity. The relatively stable profile of 3PH makes it a promising candidate for applications requiring long-term color consistency, such as self-cleaning coatings or aesthetic layers.

Over time, perceptible chromatic shifts in the CIE *a*/*b* color space—manifesting as fading, yellowing, or reddening—can degrade aesthetic quality, reducing color fidelity and lowering user acceptance in outdoor coatings and visible devices. However, such color changes do not necessarily imply loss of protective function; for many coating systems, appearance alterations have been shown not to correlate with degradation in protective performance [57]. Only in specialty contexts—such as electrochromic devices or optical color filters—can color drift indicate underlying chemical degradation of dyes or chromophores, potentially compromising both visual functionality and long-term stability. Environmentally driven photodegradation—induced by UV radiation, elevated temperature, humidity, and airborne pollutants—accelerates such shifts. Incorporating UV absorbers, hindered amine light stabilizers, or inorganic screening agents (e.g., ZnO or CeO_2_ nanoparticles) has been demonstrated to mitigate early fading and prolong colorfastness in organic coatings [58].

The CIE *a*/*b* chromaticity plot clearly places the 3PH and 3PT within the lower-left quadrant—i.e., both *a* and *b* are negative, corresponding to greenish-blue hues. Over time, these points shift modestly, indicating minor perceptual hue drift. In contrast, the P3 (purple triangles) series begins in a similar region but migrates gradually toward less negative *b* and slightly more positive coordinates, suggesting a slight yellowing or reddening trend from the original blue-green baseline. In CIELAB, *a* represents the red–green axis (negative toward green, positive toward red) and *b* the blue–yellow axis (negative toward blue, positive toward yellow). Thus, the observed drift of P3 toward less negative *b* implies a perceptual hue shift toward yellowish tones, while increases in *a* would likewise suggest a move toward redder hues. These subtle coordinate movements are consistent with minor color degradation or dye aging, but their appearance within the same quadrant indicates the dominant hue remains in the green–blue region.

The inset bar graph further confirms the photocatalysts’ performance stability by depicting the photocatalytic efficiency in MB degradation process across seven successive cycles. While the efficiency decreases slightly from initial values exceeding 95% to a range of approximately 74–83% by the seventh cycle, the catalysts retain a high level of activity throughout. This modest decline suggests minor deactivation, possibly due to surface fouling or gradual loss of active sites. Nevertheless, the sustained efficiency after multiple cycles underscores the robustness and practical reusability of the catalysts, making them suitable candidates for long-term photocatalytic applications.

The stronger color change observed for PTCA-sensitized TiO_2_ during photocatalysis can be attributed primarily to the structural features of PTCA and its mode of interaction with the semiconductor surface. PTCA possesses an extended π-conjugated aromatic core coupled with multiple carboxyl substituents. This structural configuration not only enables efficient absorption of visible light but also ensures strong anchoring of the sensitizer onto the TiO_2_ surface through stable carboxyl–titania interactions. Such anchoring promotes rapid electron injection into the conduction band of TiO_2_, which enhances photocatalytic activity but simultaneously exposes PTCA to intense oxidative conditions at the interface. As a result, the sensitizer undergoes structural transformations more readily compared to alternatives, leading to perceptible color alterations.

The degradation pathway of PTCA further explains the pronounced chromatic changes during cycling. Photocatalytic oxidation commonly involves decarboxylation processes, which disrupt the extended conjugated system of the perylene chromophore. This disruption alters the electronic transitions responsible for its characteristic absorption in the visible region, producing a brownish coloration in the case of 3PT. In contrast, other sensitizers, such as CuPcTC is a metal-complex dye having different substitution patterns, resulting in subtle spectral and chromatic modifications upon photocatalysis. Consequently, the color shift of PTCA reflects both its structural susceptibility to oxidative transformation and its enhanced interfacial reactivity with TiO_2_ under photocatalytic conditions.

## 5. Conclusions

Hybrid photocatalytic materials were developed through the formation of heterojunctions between metal oxide semiconductors, and organic semiconductor dyes, with the aim of achieving highly efficient structures for the photocatalytic degradation of pollutants in water streams. The resulting hybrid materials, prepared via the described method, integrate the advantageous properties of metal oxide semiconductors—namely, chemical, thermal, and photochemical stability—with those of organic semiconducting dyes, which exhibit broad-spectrum light absorption, efficient generation of charge carriers (electrons and holes), and tunable optical properties.

Future work may focus on molecular engineering of organic semiconducting dyes with enhanced stability, co-sensitization approaches, and integration into heterostructured photocatalytic systems to further boost performance under solar irradiation.

## Figures and Tables

**Table 1:** Composition of the reaction mass.

Sample	TiO_2_ (g)	iPrOH (mL)	CuPcTC 0.1 mM iPrOH (mL)	CuPcTC Content in TiO_2_ (%)	PTCA 0.1 mM iPrOH (mL)	PTCA Content in TiO_2_ (%)	TBAOH 40% in Water (µL)
1PH	1	20	1.3	0.1	-	-	7
2PH	1	20	2.6	0.2	-	-	14
3PH	1	20	5.2	0.4	-	-	28
1PT	1	20	-	-	2.5	0.1	7
2PT	1	20	-	-	5	0.2	14
3PT	1	20	-	-	10	0.4	28
P1	1	20	1.3	0.1	2.5	0.1	14
P2	1	20	2.6	0.2	5	0.2	28
P3	1	20	5.2	0.4	10	0.4	56

## Data Availability

The original contributions presented in this study are included in the article/Supplementary Material. Further inquiries can be directed to the corresponding author.

## Supplementary Materials

The following supporting information can be downloaded at: https://www.mdpi.com/article/10.3390/ma18204715/s1, Figure S1. Kubelka-Munk conversion of reflectance spectra; Figure S2. Tauc plots (P3, 3PH, 3PT); Figure S3. DRIFT of PTCA isolated after preparation (25 °C) and after drying at (105 °C); Figure S4. DRIFT of P3 in the range 1350–1750 cm^−1^ (A = area of the peak); Figure S5. Nyquist plots registered for the SPE, CS/SPE, CS-P3/SPE, CS-3PT/SPE and CS-3PH/SPE sensors (frequency range: 1 MHz–0.1 Hz, AC amplitude: 10 mV, OCP); Figure S6. Photocatalytic degradation of MB at the surface of P3 coatings under arc-xenon light illumination; Figure S7. UV-Vis spectra of NBT during exposure to LED light for 4 h.
