# Supplementary material for: A Dual-Sensitizer Strategy for Enhanced Photocatalysis by Coupling Perylene Tetracarboxylic Acid and Copper Phthalocyanine Tetracarboxylic Acids on TiO2
Source: Materials (Basel). 2025 Oct 14;18(20):4715. doi: 10.3390/ma18204715 (PMC12566004; doi:10.3390/ma18204715)
Supplement: Supplementary file 1 [file materials-18-04715-s001.zip › materials-3851159-supplementary.pdf]

A Dual-Sensitizer Strategy for Enhanced Photocatalysis by Coupling Perylene Tetracarboxylic Acid and Copper Phthalocyanine Tetracarboxylic Acids on TiO_2

Alina Raditoiu [1], Florentina Monica Raduly[1], Maria Grapin[1,2], Radu Claudiu Fierascu[1,2], Cristian-Andi Nicolae[1], Bogdan Trica[1] and Valentin Raditoiu[1,*]

[1] National Institute for Research & Development in Chemistry and Petrochemistry—ICECHIM,202 Splaiul Independentei, 060021 Bucharest, Romania; coloranti@icechim.ro (A.R.); monica.raduly@icechim.ro (F.M.R.); maria.grapin02@gmail.com (M.G.); fierascu.radu@icechim.ro (R.C.F.); ca_nicolae@yahoo.com (C.A.N.); bogdan.trica@icechim.ro (B.T.); vraditoiu@icechim.ro (V.R.)

[2] Faculty of Chemical Engineering and Biotechnology, National University of Science and Technology Politehnica Bucharest, 1–7 Gh. Polizu Street, 011061 Bucharest, Romania

* Correspondence: vraditoiu@icechim.ro

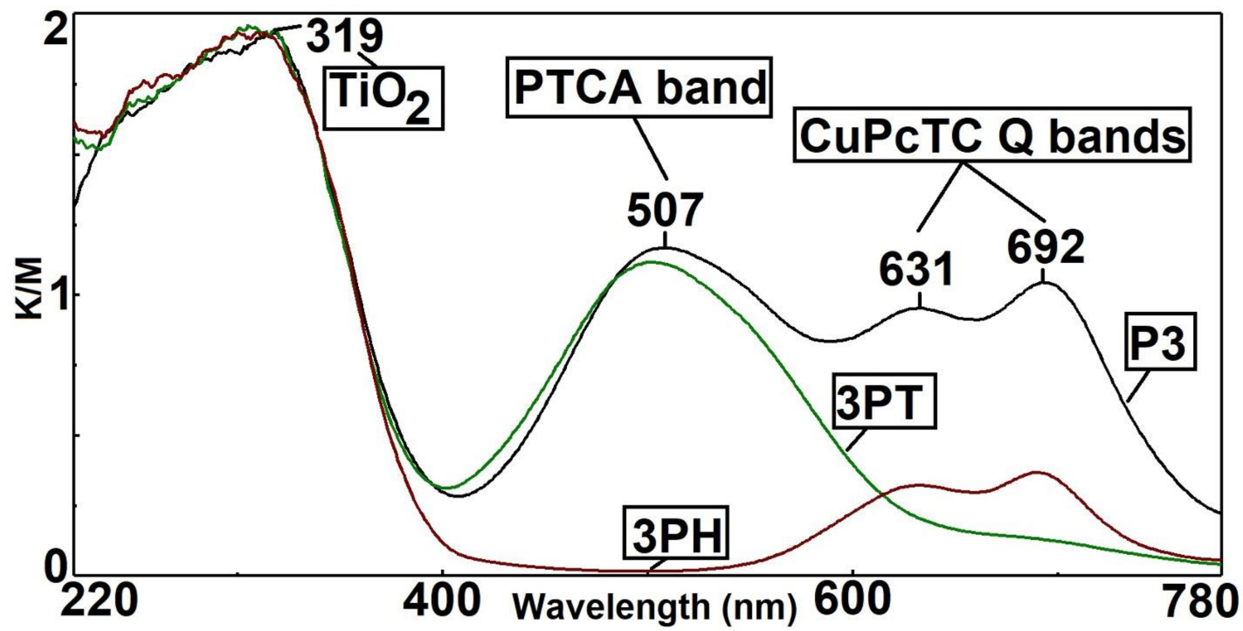

Figure S1. Kubelka-Munk conversion of relectance spectra

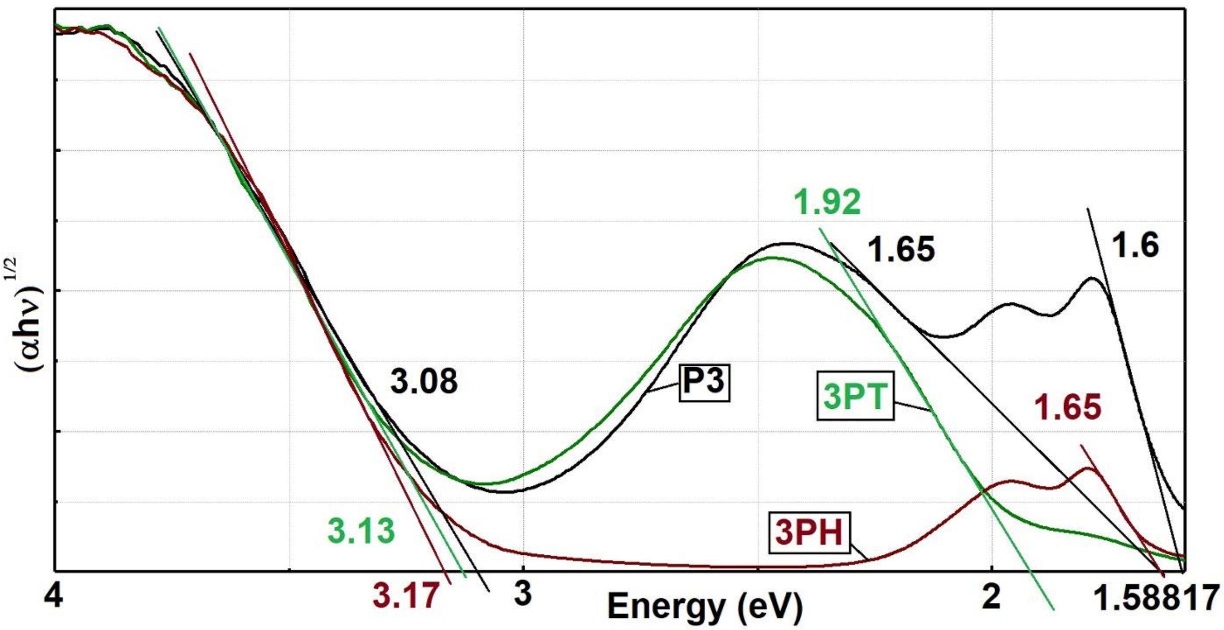

Figure S2. Tauc plots (P3,3PH,3PT)

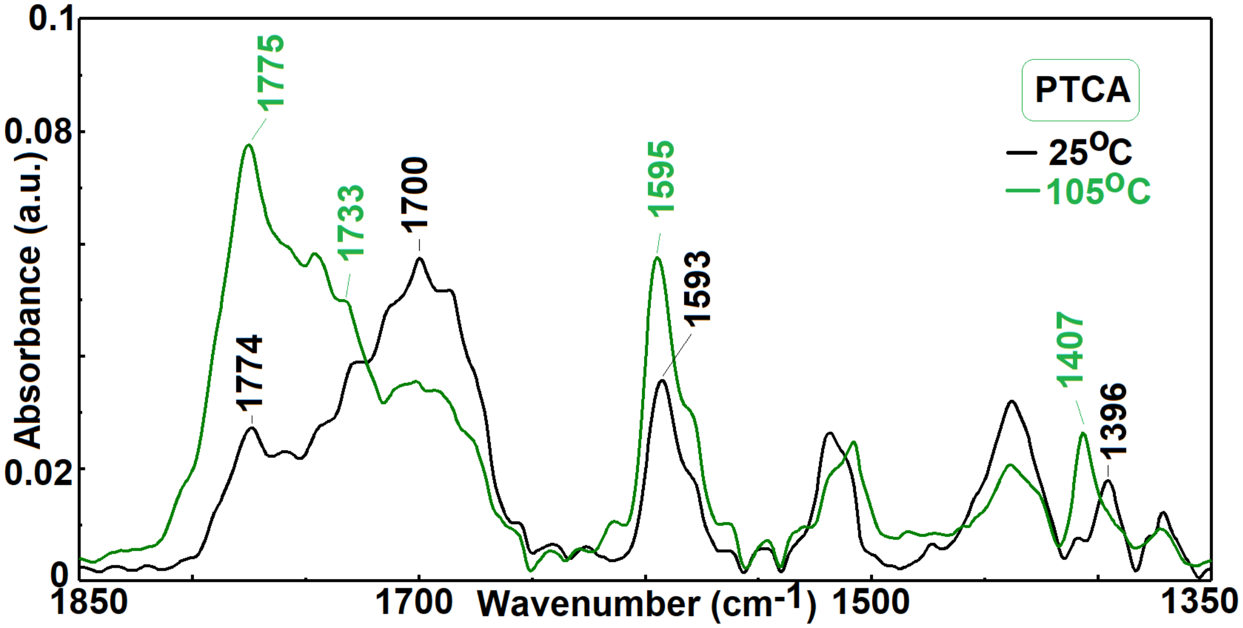

Figure S3. DRIFT of PTCA isolated after preparation (25°C) and after drying at (105°C)

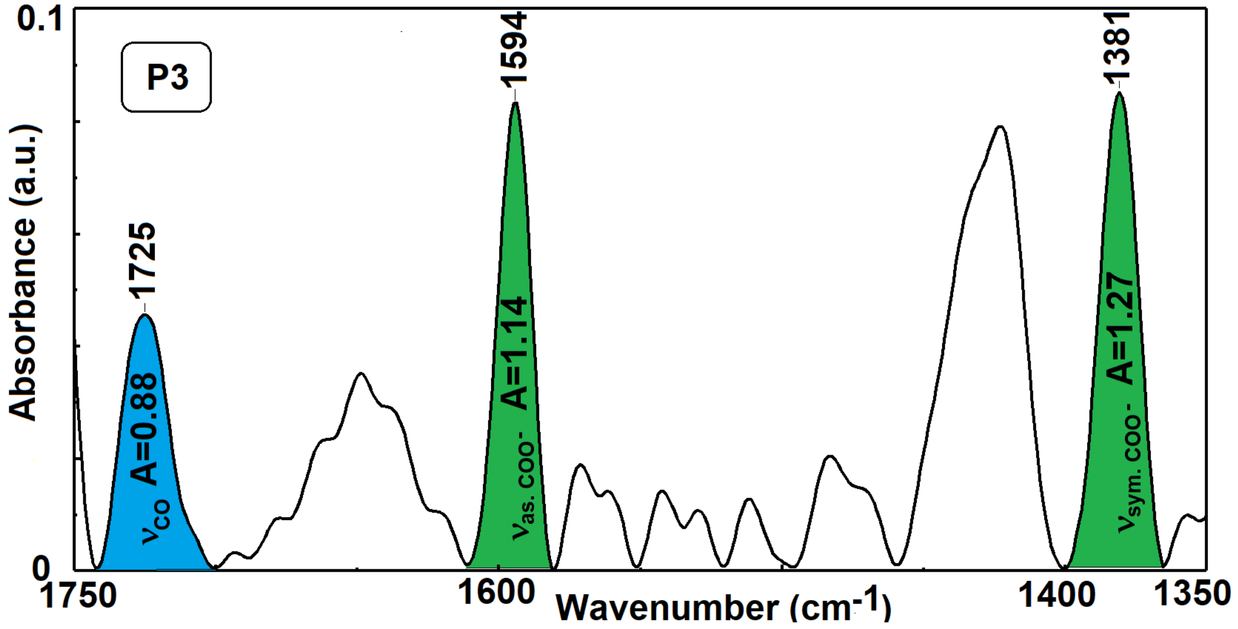

Figure S4. DRIFT of P3 in the range 1350-1750 cm^{-1} (A=area of the peak)

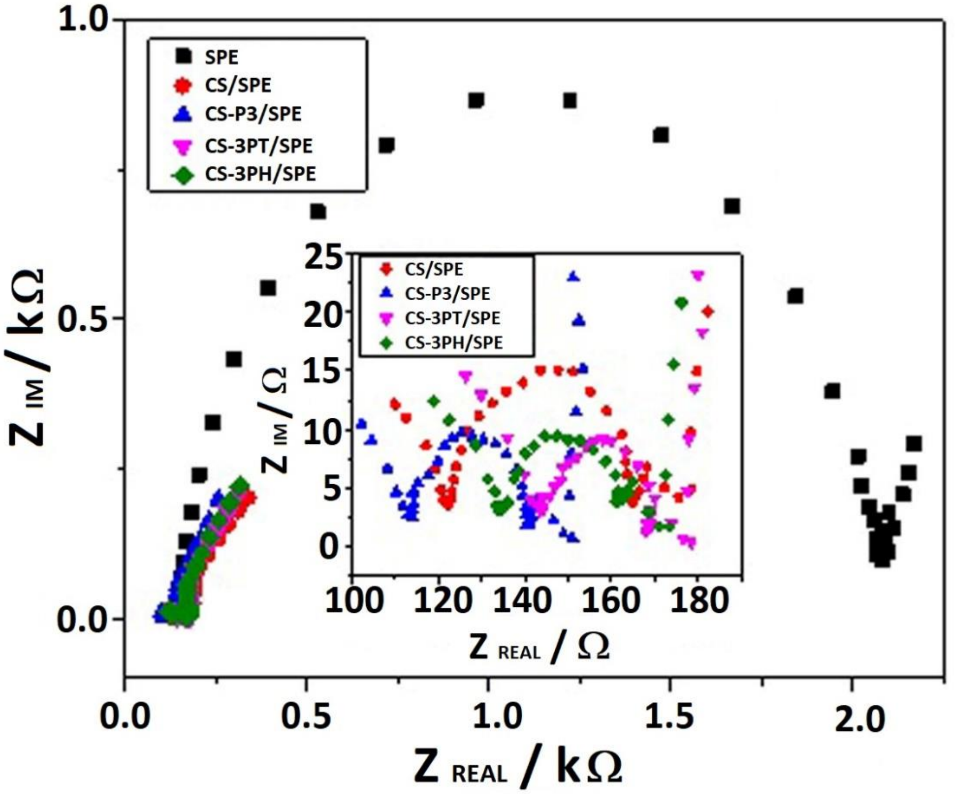

Figure S5. Nyquist plots registered for the SPE, CS/SPE, CS-P3/SPE, CS-3PT/SPE and CS-3PH /SPE sensors (frequency range: 1 MHz–0.1 Hz, AC amplitude: 10 mV, OCP).

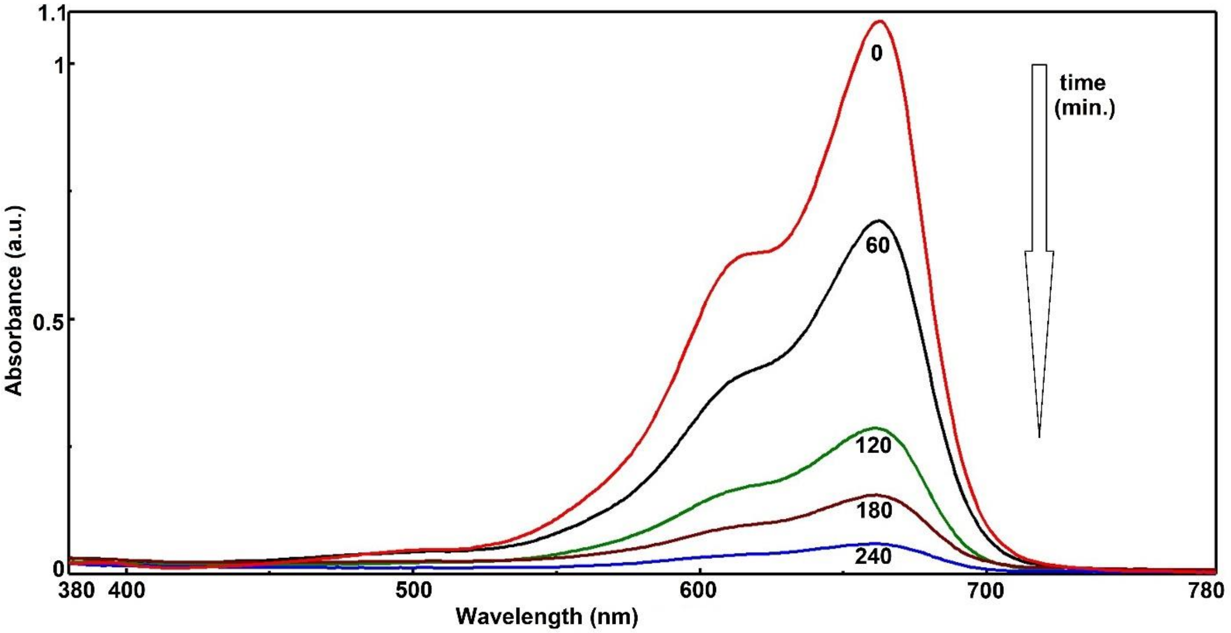

Figure S6. Photocatalytic degradation of MB at the surface of P3 coatings under arc-xenon light illumination.

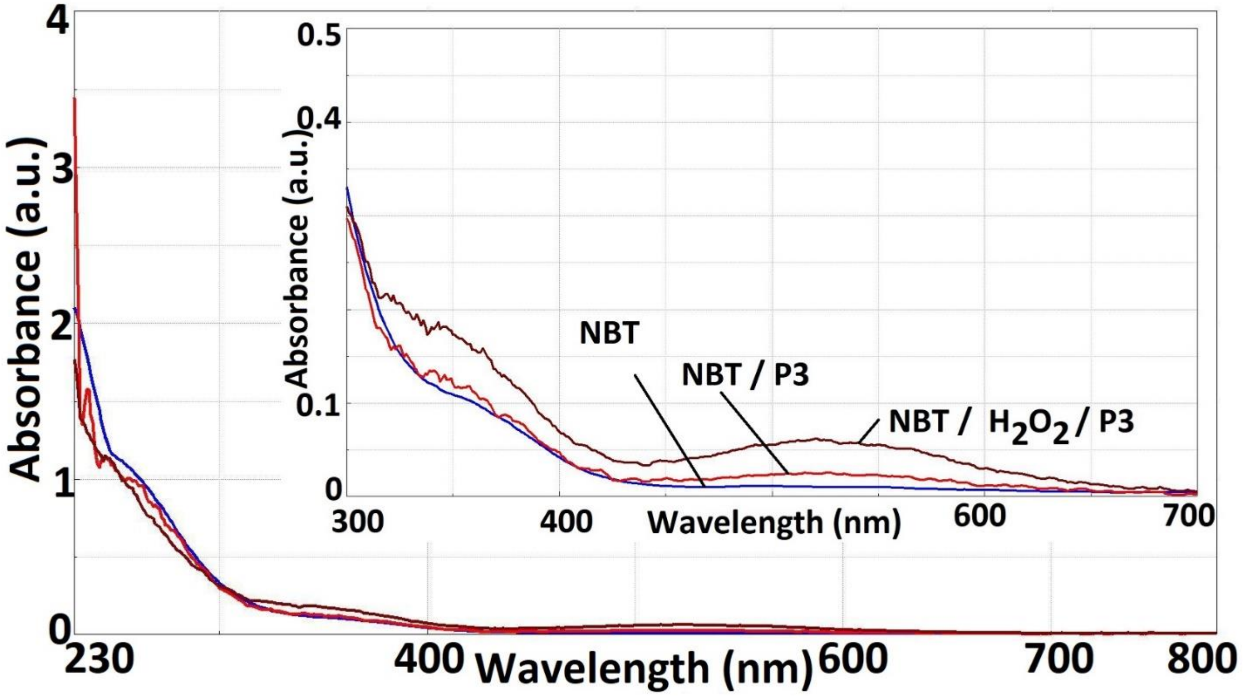

Figure S7. UV-Vis spectra of NBT during exposure to LED light for 4 h
